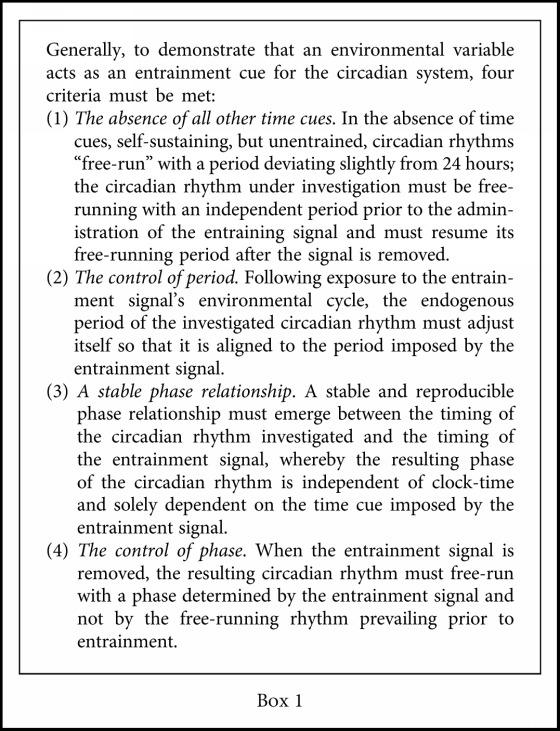# Precision Light for the Treatment of Psychiatric Disorders

**DOI:** 10.1155/2018/5868570

**Published:** 2018-01-11

**Authors:** Sevag Kaladchibachi, Fabian Fernandez

**Affiliations:** ^1^Department of Psychology, University of Arizona, Tucson, AZ, USA; ^2^Department of Neurology, University of Arizona, Tucson, AZ, USA; ^3^McKnight Brain Research Institute, University of Arizona, Tucson, AZ, USA; ^4^BIO5 Institute, University of Arizona, Tucson, AZ, USA

## Abstract

Circadian timekeeping can be reset by brief flashes of light using stimulation protocols thousands of times shorter than those previously assumed to be necessary for traditional phototherapy. These observations point to a future where flexible architectures of nanosecond-, microsecond-, and millisecond-scale light pulses are compiled to reprogram the brain's internal clock when it has been altered by psychiatric illness or advanced age. In the current review, we present a chronology of seminal experiments that established the synchronizing influence of light on the human circadian system and the efficacy of prolonged bright-light exposure for reducing symptoms associated with seasonal affective disorder. We conclude with a discussion of the different ways that precision flashes could be parlayed during sleep to effect neuroadaptive changes in brain function. This article is a contribution to a special issue on *Circadian Rhythms in Regulation of Brain Processes and Role in Psychiatric Disorders* curated by editors Shimon Amir, Karen Gamble, Oliver Stork, and Harry Pantazopoulos.

## 1. Introduction

Before we could even begin to decipher the depths of its influence on our mind and body, light loomed large in our conceptions of existence. According to the Book of Genesis, which is Judeo-Christianity's most ancient written account of the universe's origin, God's initial act of creation on the first day was the inception of light out of the void, which was separated from darkness and deemed good. If the passage is interpreted literally—as it was by many throughout history—it underscores the preeminence of light in the hierarchy of our being, placing it at the very foundation of our existence. Taken more figuratively, the placement of light at the root of the act of creation speaks to light's role as an important evolutionarily driving force for our physiology, behavior, and psyche.

The scientific evidence accumulated over the ensuing millennia has confirmed light's primacy over the temporal order of terrestrial organisms and organisms living close to or with migration routes near the ocean's surface [1, 2]. The 24-hour pattern of sunlight created by the Earth's rotation causes sweeping intraday changes in many ecosystems of the environment, characterized by cycles of illumination, temperature, and humidity and in the height and strength of ocean tides [3]. These daily geophysical changes signal reliable changes in the probability of successfully negotiating activities linked to reproductive success [4–6]. To exploit this predictive validity, multicellular animals were pressured to evolve an internal timekeeping system within the brain or other central organs that provide a *close* (but not altogether accurate) approximation of night and day length. This system was equipped with specialized photoreceptors capable of filtering out noise from the visual environment during dawn and dusk while using alterations in the amount and spectral composition of twilight to extract highly specific time-of-day information [7]. These inputs would ultimately calibrate the central pacemaker's representations of day and night—so that the frequency of the pacemaker matched the 24-hour frequency of the Earth's rotation—and phase locked its activity to align with the rising and setting of the sun [8]. With this information, the brain's clock could then use a variety of electrochemical outputs to effect internal synchronization between the central nervous system and the body's other organs and tissues, thus ensuring that macroprocesses such as metabolism and immune function are coordinated with respect to feeding and rest [9–11].

In mammals including humans, the retinohypothalamic tract (RHT) and the intergeniculate leaflet provide input pathways from photoreceptors in the retina to central pacemaker neurons of the suprachiasmatic nuclei (SCN) [12–14]. Though light operates as their chief *zeitgeber* (from the German *zeit* = time; *geber* = giver, or synchronizing agent), the SCN can interpret several exogenous and physiological time-of-day cues to entrain other satellite oscillators in the periphery and their respective signaling networks [15]. The resulting integration and transduction of these inputs into a singular circadian output enables precise homeostatic timing of diverse biological and psychological processes. The SCN achieves this in part through a multisynaptic pathway that connects it with the pineal gland and the pineal gland's secretion of melatonin [16–19], a sleep-organizing hormone or chronobiotic [20, 21]. Light processed by-way-of the SCN suppresses melatonin secretion, giving way to cycles of melatonin peaks and troughs that travel in accordance to the daily photoperiod [18]. Melatonin rhythms, in turn, facilitate cycles of arousal and offline restorative activity that are underpinned by changes in myriad biological functions such as respiration, heat generation or cooling, blood pressure, hormone production, and neurotransmitter recycling [22–24].

The central role of light in circadian entrainment is now universally acknowledged [25] and evident in hindsight, given early characterizations of the photic transduction pathway from the retina along the RHT to the SCN in rodents and primates [26]. Destabilization of temporal niches with poor indoor availability of sunlight during the day and exposure to artificial light at night is equally acknowledged for its deleterious effects on human health [27]. That said, establishing the primacy of light among other possible synchronizing cues available in the environment required that a significant (and erroneous) presupposition regarding human circadian entrainment be overcome. In the narrative that follows, we retrace the history of nonhuman and human circadian experimentation and discuss efforts made by scientists such as Czeisler et al. and Lewy et al. to show that light was a meaningful timekeeping signal that could be exploited to treat sleep-wake disturbances and subtypes of depression with seasonal or chronobiological symptoms. This history provides an informative context for understanding the significance of more recent findings suggesting that short, intermittent flashes can manufacture inordinate circadian responses relative to longer stretches of uninterrupted light. After reviewing the “flash” literature, we speculate on ways that millisecond photic stimulation could be applied in the clinic. The refinement of current phototherapy methods with respect to spectrum composition (monochromatic versus polychromatic), patterning (microfractionation of light administration versus multihour blocks of exposure), timing (during sleep versus wake), and delivery (light-emitting diodes versus fluorescent bulbs) has ushered in a new era of sophistication that demands a reconsideration of light's treatment potential in various psychiatric disorders.

## 2. Struggles to Establish Light as the Chief Zeitgeber in Humans

Although the intellectual shift caused by Darwin's theory of evolution by natural selection in the latter half of the 19th century brought on the decline of Victorian-era anthropological exceptionalism, some of its remnants survived well into the 20th century in the circadian field. Even the earliest experimental accounts of endogenously generated circadian behaviors suggested a synchronizing role for light in their function. During the late Baroque period, De Mairan [28] was the first to demonstrate that circadian rhythms could persist autonomously from the environment. In his studies, which examined the leaf movements of a heliotrope plant (*Mimosa pudica*), he observed that the plant normally opened its leaves and pedicels during the day and closed them at night and continued to do so for periods of time approximating the length of the day and night when it was moved to a room inaccessible to sunlight [28]. Seminal studies conducted over the next two centuries would continue to build on De Mairan's early insights [29], culminating in the demonstration by Augustin de Candolle that the leaf movements made by *Mimosa pudica* in constant darkness displayed a free-running periodicity just off 24 hours (~23 hours) and his suggestion that light acted as a daily resetting signal that synchronized *Mimosa pudica*'s endogenous timekeeping to a period and phase closely aligned with the solar light-dark cycle (in other words, photoentrainment; [30]).

**Figure 1:**
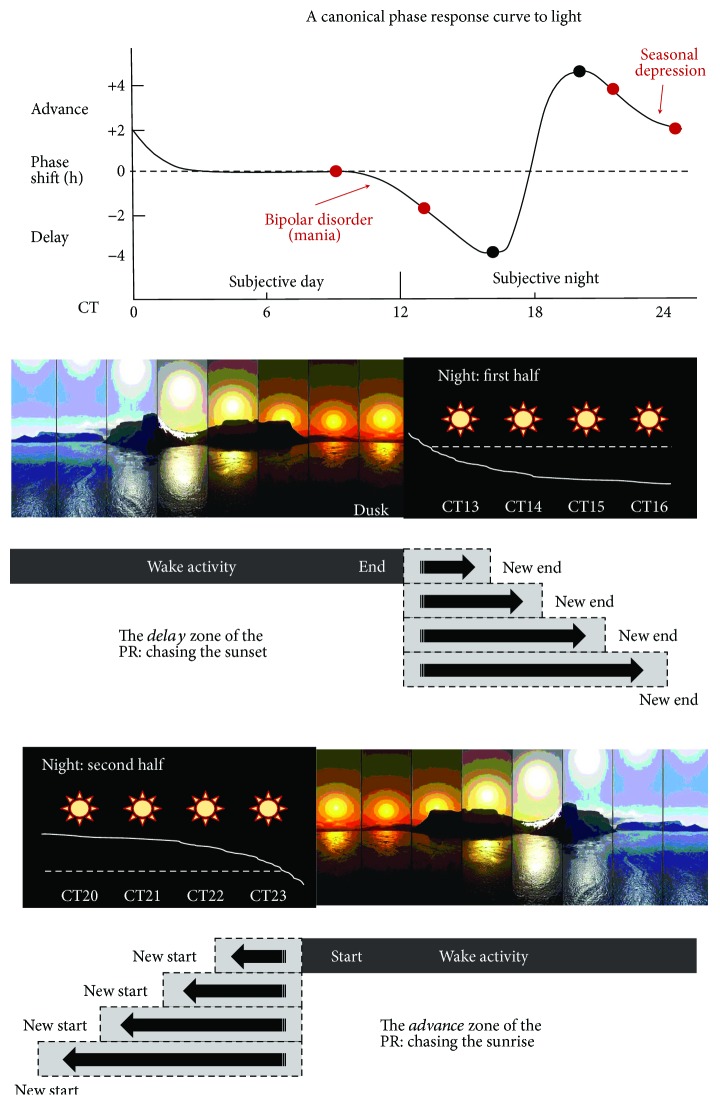
*Phase response curve to light*. The pacemaker's timekeeping responses to light are modeled by a sinusoidal PRC. Ostensibly, the PRC documents how the brain's clock shifts the body's activities so that they are always in register with the temporal beacons of sunset and sunrise. Light falling later-than-expected in the early evening is perceived as an extension of the sunset. Any significant illumination here will trigger a phase delay of a person's physiology and behavior so that they can continue to be active while the sun is still out (or perceived to still be out). On the other hand, light falling earlier-than-expected in the very late evening is perceived as the leading edge of a sunrise. Any significant illumination in this region will trigger a phase advance of a person's physiology and behavior so that they can arouse from sleep earlier to greet the sunrise (or the brain's estimate of where in the night's duration the sunrise should occur). By convention, delays in a PRC to light are plotted with negative values, while advances are plotted with positive values. In many, but not all instances, phase shifts commensurate in magnitude (hours) with the difference in timing between the photic stimulation and the onsets/offsets of a light schedule define the PRC amplitude. Shown in red are conditions whose symptoms could benefit from readjustments in circadian timekeeping. Targeting phototherapy to the shallow area of the *delay* zone can correct the advances often seen in people with bipolar disorder [87, 88]. Targeting phototherapy to the shallow area of the *advance* zone—right before a person wakes up—can offset the delays that often characterize those with seasonal affective disorder [85, 86].

By the mid-20th century, Bünning lay the intellectual seeds for our current understanding of light's effects on the circadian system and how these effects might be organized according to a temporal gate [31]. Having already documented the existence of endogenous circadian rhythms in organisms ranging from single-celled algae to humans and the genetic inheritance of the free-running period of these rhythms in plants [32–34], Bünning went on to propose that light responses that served to entrain the circadian system were *phase-dependent*. If light exposure occurred during a “tension” phase, it would delay the pacemaker by 1 to 2 hours. If, however, light was presented during a “relaxation” phase, the stimulus would advance the pacemaker's rhythm by a few hours (summarized in [35]). Subsequent work by Pittendrigh, and Hastings and Sweeney, among others, confirmed the robust phase-shifting properties of brief or prolonged light exposure on circadian functions observed in organisms across the biosphere [36–39]. However, it was a landmark paper by DeCoursey in 1960 that would set the standard for quantifying light's control over circadian timing. Her experiments were conducted with flying squirrels, which were maintained in constant darkness prior to and after delivery of a single 10 min light pulse at hourly intervals that spanned the 24-hour day [40]. DeCoursey graphically depicted the daily rhythm of sensitivity to these photic treatments, generating the first formally published phase response curve (PRC) to brief light exposure in mammals. Taking on the shape of a sinusoidal wave, the circadian PRC plots the relationship between the stimulus (light) and the measured circadian output (e.g., locomotor activity), which is delayed or advanced depending on the subjective time-of-day at which the stimulus is administered. DeCoursey found that light scheduled soon after subjective dusk produced maximal delays in the squirrel's activity rhythm, while light scheduled in the hours before subjective dawn produced maximal advances [40]. Light presented during the subjective day had no apparent influence. These two components of the PRC mapped well onto the *tension* and *relaxation* phases of circadian light sensitivity proposed by Bünning decades before, and the general shape of DeCoursey's PRC in squirrels has since been replicated in just about every organism tested to date in the laboratory ([41]; see Figure 1 for a canonical PRC to light). The evolutionary conservation of this shape almost certainly belies the central tendency of the pacemaker to preserve an animal's temporal niche [5]. It aligns the activity offset of diurnal animals with light signals that predict the end of dusk (so that the animals can remain active while the sun is still out) and activity onset with light signals that might telegraph the leading edge of dawn (so that the animals can arise earlier from sleep to greet the sunrise) [42]. The reciprocal relationship holds for nocturnal animals.

Well into the latter half of the 20th century, despite overwhelming evidence supporting the role of light as a synchronizing agent for circadian oscillations in species ranging from unicellular algae to mammals [39, 43, 44], including primates (reviewed in [45]), the capacity of light to act as a similar time cue in humans remained disputed. As late as 1980, the prevailing intellectual climate in academic circles took for granted that circadian rhythms in humans free-ran in an environment devoid of periodic time cues. However, *uniquely in all the kingdoms of life*, they were thought to be relatively insensitive to light. Social interactions, instead, were considered the driving force responsible for entraining the endogenous circadian machinery (see Box 1 for a quick review of the criteria that must be met for a stimulus to serve as an entrainment cue). Ironically, the perpetuation of this misunderstanding was the byproduct of confounding studies done in part by Aschoff, who, along with Bünning and Pittendrigh, has long been seen as one of the three most influential scientists in the circadian field. Aschoff, himself, coined the term *zeitgeber* [46], which is now ubiquitously used in the circadian literature to describe an entraining agent or time cue.

In the early 1960s, two groups independently set out to determine the presence of free-running circadian rhythms in humans. The first group, consisting of Aschoff and Wever, conducted their studies on subjects individually housed within a sealed cellar below Munich Hospital for 8–19 days [47], while Siffre, a renowned underground cave explorer, subjected himself to a two-month-long inhabitation of the underground Scarasson cavern (an ordeal later recounted in his book titled *Beyond* Time; [48]). Both accounts reported an endogenous free-running cycle of rest-activity with a period greater than 24 hours (~25 hours), suggesting that periodically occurring stimuli in the environment somehow reentrained the human pacemaker to an exact 24-hour schedule on a daily basis as they did in other animals. Initially, presuming (to their credit) that light-dark cycles would be especially important entrainment signals for people, Aschoff and Wever launched the first systematic investigation of human-relevant zeitgebers in a newly-built underground soundproof bunker specifically designed to insulate subjects from all external time cues. Their initial observations, here, suggested that an imposed light-dark (LD) cycle was a potent entrainment mechanism [49]. However, during one unfortunate experiment, an equipment malfunction resulted in the administration of the imposed LD cycle without activation of a system of gong sounds that they had set up to awaken subjects for periodic urine collection, which along with other measures (including sleep-wake behavior and body temperature) contributed to the assessment of circadian entrainment. To his surprise, Wever found that in the absence of the gong sounds the imposed LD cycle was not sufficient to entrain the subjects in question to the corresponding 24-hour day and that synchronization occurred only in experiments where the imposed LD cycle and the gong system were used in tandem [50]. Wever ultimately concluded that since the determining factor—the gong—was being interpreted by the subjects as a social contact with the experimenter, social cues must be more important zeitgebers for humans than LD cycles [50].

Six months after Wever's article appeared in the *European Journal of Physiology*, Aschoff and his associates' own study was published in *Science*, supporting and building on Wever's findings and stating that social cues were sufficient for the entrainment of human circadian rhythms [51]. The crucial flaw in their conclusion was the assumption that the social cue represented by the sounding of the gong had no corollary zeitgebers contributing to the perceived entraining effects. Unfortunately, Aschoff and Wever's protocol did not ensure the degree of light insulation that their conclusion required. In the subjects' living quarters, daytime was delineated by ceiling-mounted fluorescent lamps controlled by the experimenters. This overhead lighting was turned on at subjective dawn and turned off at subjective dusk, allowing the scientists to have total control over the LD cycle imposed on the subjects—*provided that the subjects themselves had no independent access to ectopic sources of light*. This was not the case. The subjects were given free access to lower-intensity kitchen, bathroom, bedside, and desktop lamps throughout the evaluation. If these lamps were used by the subjects every time the gong went off during the experimenter-imposed dark period, the *bona fide* entrainment effects caused by the lower-intensity light could be erroneously attributed to the gong.

Despite the resultant view that pervaded the 1970s, a number of scientists remained convinced that light was an important entrainment cue for the human circadian system and carried out their own investigations. These groundbreaking studies, which are discussed in the section below, helped reorient the prevailing entrainment paradigm for humans and restored light to its rightful place atop all circadian zeitgebers. In the clinical realm, these studies also prompted the psychiatry field to take a closer look at a newly emerging tool whose utility was being explored for the treatment of mood disorders.

## 3. Let There Be Light

Whereas Aschoff and Wever's studies permitted *ad libitum* use of personal lighting, Czeisler and his associates tested the role of the LD cycle as a time cue in humans using an experimental design analogous to that employed in most animal studies showing effective circadian photoentrainment. Here, an absolute LD cycle was imposed on the subjects, eliminating all secondary illumination and creating consolidated alternating intervals of bright light and near darkness. In this study [52], which was conducted at the Montefiore Hospital Laboratory of human chronophysiology, two young twentysomething males living in isolated apartments were allowed to self-select times of sleep, meals, and lighting for 25 days. Over the course of about one month, the subjects typically free-ran with a period of ~25 hours. The subjects were then exposed to a 24-hour LD cycle with firm transitions between dawn and dusk for 9 days while being deprived control over any other light-emitting devices. After a few transients (transition days), they became entrained to the imposed 24-hour light schedule, before free-running again upon release from the LD cycle into a zeitgeber-free environment with *ad libitum* access to food, sleep, and personal lighting. Importantly, the subjects began to free-run with a phase predicted by the LD cycle rather than the phase predicted by the preexisting free-running period that had prevailed in the first 25 days of the experiment (i.e., an entrainment criterion). Based on these findings, Czeisler and his colleagues concluded that an LD cycle alone could be an effective synchronizer of the human circadian system whether social contacts were available or not. To solidify their conclusion, the research team went on to demonstrate that repeated exposure to 4 hours of bright light (~9500 lux) at a circadian phase coinciding with subjective dusk causes a rapid and stable several-hour phase delay in body temperature and cortisol rhythms, independent of the subject's sleep/wake cycle [53].

By the late-1980s, in the 30 years that followed DeCoursey's seminal report, PRCs to light had been reported in all eukaryotes studied except for man. Having shown the synchronizing effect of light in a single elderly subject exposed to bright light [53], Czeisler and his associates expanded their sample size and—over the course of 45 individual experiments—examined the phase responses of the human circadian clock to bright (~9500 lux) light at various times of the 24-hour day. Using core body temperature as a circadian phase marker, their work [54], in conjunction with a more obscure study done by K. Honma and S. Honma [55], provided the field with the first quantitative human PRC to light. In addition to demonstrating that the response of the human circadian pacemaker to light is well within the range of sensitivity observed in lower organisms, Czeisler et al. also challenged the contemporaneous belief that human circadian timekeeping was not impacted by exposure to ordinary room light. In an auxiliary analysis, the results of 23 resetting trials in which the subjects' treatment with bright light (~9500 lux, 5 hours) had occurred midway between an 11-hour block of room light (~150 lux) were compared to trials where bright-light treatment was either *preceded* or *followed up* by 11 hours of room light. The circadian phase at which bright light was administered was controlled for across these conditions. Nevertheless, the researchers found that the timing of exposure to room light (~150 lux) could affect the magnitude and direction of phase shifts induced by the bright-light regimen, suggesting that the photic sensitivity of the human circadian pacemaker extended down to at least 150 lux and was far greater than had been recognized up to that point.

Having retreated from his earlier position that changes in light intensity produced no observable effects on free-running circadian rhythms [50, 56], Wever once again found himself on the wrong side of a scientific debate by maintaining that exposure to light above 2500 lux was required to exert a direct effect on the human circadian pacemaker, such that any effects observed after stimulation with less than 1500 lux could be attributed to behavioral factors alone [57, 58]. Two converging lines of evidence steadily contradicted this notion. First, based on the ancillary analysis of their human PRC data, Czeisler and his associates began to explore the human circadian pacemaker's lower range of light sensitivity. Nine young male subjects participated in a 16-day study during which light treatment consisted of multiple 5-hour exposures to moderately bright light of approximately 1260 lux [59] or lower-intensity light at 180 lux [60]. After an initial phase assessment period, the 5-hour exposures were timed so that they were centered 1.5 hours after the endogenous temperature minimum (late night/early morning). Based on mathematical modeling of light's drive on the human circadian pacemaker by Kronauer [61, 62], the researchers predicted that light scheduled at this phase would cause an advance in the subjects' core body temperature rhythm. As predicted, 180 lux of light produced a significant phase advance of temperature rhythms; moreover, the advance achieved was almost 50% of the shift observed with 1260 lux [59, 60]. Similar circadian photosensitivity has been shown to occur in the delay zone with the delivery of 100-lux light over 6.5 hours [63].

The second line of evidence suggesting that the human circadian system was responsive to moderate intensity light concerned a separate corpus of work on melatonin that also struggled to overcome the idea that, alone in the animal kingdom, evolution had conferred in humans an ability to escape the biological controls imposed by sunlight. Melatonin, one of the principal organizers of the sleep/wake cycle, was first isolated from bovine pineal glands by Lerner et al. in the late 1950s [64, 65]. Soon, in both diurnal and nocturnal animals, its production from the pineal gland was found to be low during the day and restricted predominantly to the nighttime [66, 67]. These observations across temporal niches hinted at a role for ambient light in shaping the brain's melatonin regulation. On the heels of a demonstration in rodents that melatonin secretion was, indeed, inhibited by light [68, 69], studies in numerous other mammalian species confirmed that the hormone is gated by exposure to artificial or natural lighting and that its major release period is closely linked to the evening: invariably, the onset of melatonin secretion coincides with sunset and its offset with sunrise (reviewed in [70]).

The circadian rhythm resulting from the relationship between light and melatonin secretion was shown to free-run in superfused avian pineal glands [71], suggesting that melatonin rhythms—at least in birds—were a direct output of the endogenous clock machinery and not (only) a passive response to photic stimulation. These observations set the stage for melatonin's eventual use as a reliable marker for circadian phase in humans (so-called “dim light melatonin onset or DLMO”) [72]. However, just as the human circadian pacemaker was thought to be insensitive to resetting by light, it was generally accepted throughout the 1970s that human melatonin production was similarly unaffected [73–80]. A sea change in this outlook started in 1978 when for the first time Wetterberg and a separate team at the National Institute of Mental Health (NIMH) led by Lewy et al. reported that bright-light exposure at night could block melatonin secretion in healthy subjects, as well as those with various medical conditions [81, 82]. Later studies continued to lower the bar for the minimum intensity of light considered necessary for curtailing acute melatonin secretion. Estimates now suggest that significant reductions are possible in humans with as little as 200–300 lux [63, 83].

## 4. Light as a Therapeutic Tool

Within the blink of an eye in 1980-1981, Czeisler et al. and Lewy et al. had quickly provided two lines of evidence showing that physiologically relevant levels of light exposure could produce measurable responses in human brain function. Since humans likely had many biological rhythms that were entrained to the terrestrial LD cycle, Lewy and his associates theorized that bright artificial light could be used experimentally to manipulate these rhythms for therapeutic ends and particularly so in psychiatric disorders. They first tested this possibility in a patient whose yearly bouts with depression coincided with the shortened day lengths of winter and receded with the onset of spring [84]. The researchers initially hypothesized that this seasonal rhythm was determined by the patient's truncated photoperiod and that by extending the length of the day with bright artificial light at dawn (between 6 am and 9 am) and dusk (between 4 pm and 7 pm), they might rectify his winter depression. This was, in fact, the case after 4 days of exposure to 2000-lux light scheduled as described [84]. Despite their initial hypothesis about day length, Lewy et al. eventually settled on the proposal that the observed antidepressant effects of light were predicated on a synthetic correction of abnormally phased circadian rhythms (i.e., the “phase shift hypothesis or PSH;” [85, 86]). From this perspective, they reasoned that many mood disorders could be reenvisioned as chronobiological disorders where the phase of the endogenous circadian system was mismatched with respect to real-time and one's sleep schedule. Bright-light administration could bring this system back into balance. It is worth noting that the PSH model was likely influenced by the earlier thinking and work of Kripke and his associates, who provided some of the first evidence that (1) the circadian clock of a patient meeting diagnostic criteria for bipolar disorder was accelerated relative to that of neurotypical individuals, hampering the patient's synchronization with the 24-hour day, and (2) the therapeutic effects of lithium in this condition might derive from the drug's circadian phase-delaying properties [87, 88]. Kripke et al. were some of the first active investigators of bright-light therapy in mood disorders [89] and, along with Wehr et al. at NIMH [90, 91], were contemporaries of Lewy et al. and their efforts to establish the treatment potential of scheduled bright-light exposure.

According to the PSH, individuals suffering from winter depression, or *seasonal affective disorder* (SAD) as it is commonly referred to today [92], were hypothesized to suffer mostly from abnormal delays in circadian timing [85, 86]. The majority would therefore preferentially respond to morning bright light, which—assuming a human PRC to light—would provide a corrective phase advance. The PSH also proposed the existence of a minor subgroup of SAD patients whose circadian rhythms were abnormally phase-advanced and who would benefit from the delays in timing that would come with evening bright-light exposure. Providing support for this model, Lewy and colleagues found that out of 8 SAD patients tested, 7 preferentially responded to the antidepressant effects of morning bright-light administration and did so with corresponding advances in their DLMO [93]. Only one patient was found to preferentially respond to evening light with a corresponding DLMO delay (*ibid*). Together, these preliminary findings argued that mood disorders had circadian underpinnings and could be treated or supplemented with the timed delivery of light. The findings would spawn a multitude of clinical trials examining the efficacy of bright-light therapy. In addition to SAD [94–99], studies have looked at bright light's effects on other mood disorders including nonseasonal forms of depression such as bipolar disorder and major depressive disorder (reviewed in [100–102]), as well as sleep disorders (reviewed in [103, 104]) and neurodegenerative diseases, such as Parkinson's [105, 106] and Alzheimer's [107].

The 1990s were met with a larger wave of success stories that accompanied several investigations of light's antidepressant efficacy in those suffering from SAD. In an aggregated sample of more than 300 subjects living across the United States at the same Northerly latitude, teams led by Terman et al. (Columbia University, New York, 41° N), Eastman et al. (Rush Medical Center, 42° N), and Lewy et al. (Oregon Health Sciences, 45° N) showed that scheduled morning exposure to 2500–10,000 lux of cool-white fluorescent light over 2 weeks could reduce behavioral ratings of depression relative to placebo [108–110]. These reductions were sufficiently large to meet remission criteria in upwards of 30–60% of the patients tested, a feat rarely observed even in large clinical trials of antidepressant drugs such as Prozac [111]. The Terman-Eastman-Lewy studies were published back-to-back in the *Archives of General Psychiatry*. The rigor of their studies—and the visibility that came with their publication venue—inched the psychiatry field closer towards formally recognizing light's utility as a therapy or therapeutic adjunct in mood disorders [112]. However, later meta-analyses of scheduled bright-light exposure in seasonal and nonseasonal depression would prove ambivalent in their support of light's clinical efficacy. At least five meta-analyses, including two compiled for the Cochrane Database of Systematic Reviews, have questioned the statistical relevance of the phototherapeutic effects reported due to risks of bias in patient selection, small sample sizes, limited use of placebo controls, allowance of patient self-rating of outcome measures, and lack of checks on treatment compliance [96, 98, 99, 113, 114]. A less charitable interpretation would suggest that the bulk of scientific literature that has accumulated in support of bright-light treatment is built atop a sandcastle foundation: any results must be interpreted with the utmost caution due to, more often than not, flawed experimental design. And yet, randomized and double-blind clinical trials to the present day continue to suggest the utility of scheduled light exposure for symptom mitigation in SAD [115–118]. This stream of peer-reviewed study has reached a point where Medicare and most insurance companies have deemed it medically appropriate to treat patients who meet diagnostic criteria for SAD with high-intensity light boxes capable of emitting 10,000 lux [119, 120]. Arguably, in the final analysis, this recognition has come at the expense of a wider recognition that light treatment has a therapeutic value in other affective disorders and might have unexplored potential to address various symptoms associated with other conditions listed across the DSM-V (*Diagnostic and Statistical Manual of Mental Disorders, Fifth Edition*), including neurodevelopmental, neurocognitive, eating, and substance abuse disorders.

Among possible culprits, it is more likely than not that patient compliance is one significant factor that has historically weighed down estimates of light's efficacy when exposure has been carefully timed to a subject's endogenous circadian phase. The reasons for this are not difficult to understand. The standard treatment approach that has emerged with “bright-light therapy” involves the use of a fluorescent ballast that produces diffuse white light within a few feet of the subject's eyes. Administration is timed within the morning as close to waking as possible [121]. An oft-cited dose-response curve has become universally accepted for antidepressant action. It has a defined threshold of 5000 lux, which can be achieved with a threshold dose of 2500 lux over two hours, 5000 lux over one hour, or 10,000 lux for a minimum exposure period of 30 min (the de facto gold standard protocol given most healthcare insurance policies). Thus, for a commitment of at least half an hour at a fixed time each morning, a person on a light regimen must sit relatively still in front of an uncomfortably bright lamp while the demands of an early morning schedule (e.g., making breakfast, preparing for a commute to work, and getting children ready for school) go on about them. The person must do this with steady discipline on weekdays with an ever-changing social calendar, as well as on weekends and holidays. What is more, they are encouraged to do so even when experiencing temporary unpleasant effects such as headache, eye strain, nausea, or jitteriness [122, 123].

In retrospect, the two decades' worth of misunderstandings perpetuated by Wever and others have left long-term psychological scars in the circadian and psychiatry research fields with respect to light's dynamic range of action in the human brain. Many assume—even today—that patients require extended periods of bombardment with high-intensity, broad-spectrum light to elicit any desired therapeutic changes. This viewpoint is only now evolving with our growing understanding of mammalian circadian photoreception in the retina and the important role of melanopsin [124], which is a short-wavelength (blue) sensitive opsin expressed by a subset of retinal ganglion cells that project directly to the SCN via the RHT. Cells bearing melanopsin are innately photosensitive [125] but also receive inputs from rods and cones. Coordination of signaling between the three leads to stable circadian photoentrainment and phase-shifting responses to light exposure (though the precise logic for how this occurs remains poorly defined in humans as does the extent of connectivity between rods/cones and melanopsin-containing cells; [126–129]). The fact that the action spectra for melatonin suppression and phase resetting in humans do peak with blue wavelengths (446–480 nm; [130–132]) has led a few researchers to conjecture that melanopsin might help to mediate the antidepressant effects of light in SAD. Several investigations have explored this possibility, finding that treatment with narrowband blue light-emitting diodes (LEDs), blue-enriched white LEDs, or high color temperature lamps reduces depressive ratings on the SIGH-SAD (*structured interview guide for the Hamilton depression rating scale, seasonal affective disorders*) to an extent similarly observed after exposure to 10,000 lux broadband fluorescent light [133–136]. These reductions could be achieved with perceived illuminances between 100 and 1000 lux.

Further inquiries have been made into whether physiological melanopsin responses are different in individuals suffering from depression or whether preexisting variants in the melanopsin gene (*OPN4*) might mediate depression risk. Available data suggest that the melanopsin-mediated pupillary constrictions that occur postillumination are, in fact, diminished in people suffering from either major depressive disorder or SAD [137, 138]. A specific coding variant of *OPN4* (P10L) resulting from a single nucleotide polymorphism in exon 1 (rs2675703), a sequence corresponding to the N-terminal tail of the melanopsin protein, has also been shown to segregate more in samples of patients with SAD compared to control samples [139]. These findings suggest that differences in the nonvisual circadian system may very well predispose some individuals to mood disorders and affect their responses to light treatment.

From a larger perspective, the intersection of the circadian photoreception and depression literatures that occurred throughout the 2000s began to provide a roadmap for how to escape the “hammer” approach that has long dominated phototherapy practice. As of 2010, regimens making use of lower-intensity light administration with smaller devices capable of producing specific wavelength emissions could be entertained as next-generation treatment strategies with more antidepressant efficacy and fewer compliance issues. However, another basic discovery in the human circadian field in the last several years foretells of even greater possibilities for how we might soon use light to improve mental health. In the last section of this review, we summarize recent findings showing that millisecond sequences of light can trigger inordinate circadian phase shifts relative to continuous exposure and speculate on the various ways this phenomenon can be harnessed to develop patient-oriented phototherapies. The computational space that flash exposure offers, combined with the more sophisticated control mechanisms of light administration promised by LEDs, reopens the discussion on what psychiatric conditions would benefit therapeutically from ocular photic stimulation. Considering the interconnectivity of the RHT and SCN with centers in the brain that manage information and emotion [14, 140], precision treatments with flash LED exposure have the potential to cast a wide net.

## 5. Precision Light: The Future of Phototherapy

In an uncanny coincidence, two competing visions for how light is processed by the circadian system were published to varying receptions in 1984. The first, published in the esteemed journal *Nature* by Takahashi and colleagues, introduced the concept of “circadian reciprocity,” the idea, now widely held, that the size of a circadian phase shift in response to light is derived simply enough from just the intensity and duration of the light exposure [141]. Under this model, the SCN is considered nothing more than a graded photon counter: the greater the number of photons registered over a defined period in the subjective night, the greater the resulting phase shift that should be observed in an animal's physiology and behavior up to some saturation level [141–144]. The second vision, published more obscurely as a rapid communication in the *Journal of Experimental Zoology* by the noted Indian chronobiologists Joshi and Chandrashekaran [145], showed that a single bright flash of submillisecond light delivered via a Metz mecablitz flashgun could produce significant advances and delays (30–60 min) in the flight activity of the Schneider's roundleaf bat, *Hipposideros speoris* [145]. Joshi and Chandrashekaran would go on to publish a series of PRCs to light pulses of varying durations from 0.083 to 3.33 milliseconds (ms) soon after [146]. At all these durations, the pulses engineered phase shifts in *Hipposideros speoris* comparable in magnitude to 15 min of continuous illumination with 1000-lux incandescent or fluorescence light (*ibid*). The observation that very short perturbations of light approaching 1/2000 s in duration could reset the circadian clock was not completely novel; Bruce et al. had demonstrated this decades earlier both in the sporulation rhythms of the fungus *Pilobolus sphaerosporus* [44] and in the eclosion rhythm of *Drosophila pseudoobscura* [39]. However, the results in bats proved that these exposure periods were also relevant for mammals and not just an interesting phenomenon consigned to lower organisms.

It was not until 1998 that investigators would test and expand on these results in mice, rats, and hamsters, animal models with a considerably larger following in biomedical research. Using studio-grade xenon flashtubes (Dyna-Lite Flash Head), Van den Pol et al. found that a train of 2 ms pulses delivered every 1 or 5 s for 5 min, or on the minute for an hour, caused multihour phase delays in mouse running wheel activity [147]. The magnitude of this response approximated the maximal shifts in running wheel activity that are typically seen in this species after 10–15 min of uninterrupted light exposure [148]. Arvanitogiannis and Amir showed that even briefer flashes, 10 *μ*s in length, could also reset the clock in rats and do so with a combinatorial logic that integrated the responses of these flashes with shorter and longer episodes of light [149]. As few as five 10 *μ*s flashes generated from a grass stimulator system could induce behavioral and cellular correlates of clock resetting (*ibid*). Vidal and Morin have provided the most in-depth characterization published thus far on the effects of millisecond light exposure on the mammalian circadian system [150]. Probing the advance zone of Syrian hamsters (*Mesocricetus auratus*) with the same Dyna-Lite Flash equipment that Van den Pol et al. had used several years before, the researchers discovered that just ten 2 ms pulses—that is, a total stimulus package lasting 20 ms—could establish maximal drive on the circadian pacemaker. Interestingly, the efficacy of this pulse train was influenced by the rest interval between the flashes. Advances in hamster wheel running were optimized when the interstimulus interval reached 4–8 s but were impaired with quicker turnover; animals, receiving ten 2 ms pulses each separated by 0.5 s, for instance, mounted a very weak phase response or none at all [150].

The nonvisual circadian system appears deceptively simple to most outside observers. One could argue that this perception has been abetted by concepts such as reciprocity and the notion underlying it that the hardware in the brain that most determines circadian responsivity are the photosensors themselves in the retina. The more light the retina “sees,” the more this message is couriered to the SCN and the bigger the phase shift that results. This outlook has been emboldened since the discovery of melanopsin and the meticulous dissection of melanopsin-containing retinal ganglion cell pathways that has ensued since the early 2000s. This outlook, perhaps unwittingly, has reduced the perceived role of the SCN and its 16,000-neuron strong clock network to that of a passive engine in photoentrainment when, in reality, this nexus sits at the crossroads of a much more deliberative body that spans known (and unknown) specializations in the retina, the intergeniculate leaflet, and the complex circuits that interconnect them [14, 151]. Carefully peeling back the layers of flash data that have been compiled in rodents, one would be hard-pressed to conclude that photon counting is the sole mechanism by which phase shifts are calculated. A priori, this makes sense. The natural changes that occur in ambient illumination during twilight progressions between day and night involve changes in both quality (spectral composition) and quantity (intensity, probability of exposure). While waning or ramping light intensity is the most conspicuous change that accompanies dusk and dawn, respectively, we have lost sight of the fact that these illumination differences present just the last step of the estimated 30–60 min twilight progression. For the bulk of this period, the photic information that signals to the brain that day is giving way to night (or vice-versa) concerns grades of color temperature. Under the continuous daylight of the high arctic summer, daily oscillations of color temperature suffice as powerful synchronizers of avian locomotor activity [152, 153]. Recent laboratory experiments have demonstrated the synchronizing effects of photoperiod cycling every 12 hours between two different wavelength-enriched lights as well [154, 155]. Let us step back a moment to consider what these data are really telling us: they provocatively suggest that light intensity changes are *expendable* (i.e., not absolutely necessary) for circadian photoentrainment, rendering the reciprocity hypothesis nonsensical. Do these data invalidate the reciprocity hypothesis altogether? Of course not. However, they make plain that the nonvisual circadian system factors in more than just photons when engineering phase shifts that will realign endogenous rhythms with the solar day. This is the proper context for the flash experiments that were started with Pittendrigh, kept alive by Joshi and Chandrashekaran, and then bequeathed to Van Den Pol et al., Arvanitogiannis and Amir, and Vidal and Morin: we do not understand the computations that the clock network is making and, not surprisingly, just as in other systems like the trisynaptic circuits of the hippocampus, the content, duration, frequency schedule, and overall pattern with which information is sent matters.

Judging by the number of citations accrued over the past decade (all <30), articles documenting flash stimulation of circadian phase-shifting have not received a great deal of attention from the basic circadian research community. The lack of visibility of this literature has not deterred study in humans, however. In a string of experiments that started around 2010, Zeitzer et al. showed that the human circadian system has the capacity to respond to 2 ms pulses of broadband light delivered either once (473 lux, tungsten lamp) or twice (3000 lux, xenon lamp) a minute for an hour [156, 157]. The integration of these hour-long light sequences delayed rhythms in salivary melatonin by 30–45 min in flash-treated subjects, while subjects left in the dark exhibited no net phase change (*ibid*). Zeitzer et al. also made the unique observation that these flash protocols could still exert their circadian effects in people as they lay asleep, doing so without influencing alertness, sleep architecture, or state transitions between nonrapid eye movement (NREM) and REM sleep [157, 158]. The electroencephalogram (EEG) spectrum measured from C3/C4/O1/O2 remained unaffected when comparing EEG signals recorded during the photic stimulation to the signals recorded an hour before, with spectral power conserved in all the major frequency bands [157]. That flash stimuli maintain their circadian efficacy during sleep harkened back to another dynamic strategy of sleep-time light administration that was developed by Terman et al. in the late 1980s [159]. This strategy, called dawn simulation, presents a gradually rising light signal that starts dim (starlight illumination) and continues to brighten along the trajectory of a protracted sunrise until reaching ~250 lux, where it attenuates around a subject's habitual wakeup. Much of the CPU-controlled treatment occurs in the last 2-3 hours of the subject's slumber. Though dawn simulation was never widely adopted, both Terman et al. and Avery et al. found evidence for its antidepressant efficacy in several cohorts with SAD [159–165].

What is especially remarkable about the human flash studies is that they opened the door to the idea that light's phase-shifting properties could be dissociated from its ability to suppress melatonin. In two separate cohorts totaling ~40 subjects, Zeitzer et al. found that millisecond patterns of 2000–3000 lux photic stimulation that triggered phase shifts of DLMO did not influence the overall salivary concentration of melatonin, unlike continuous light exposure at the same intensity, which led to 50% reductions in hormone secretion [156, 158]. Similar to Vidal and Morin's results in rats, Zeitzer and Najjar described a phase shift logic of millisecond pulsing where circadian drive was maximized with interstimulus intervals ranging from about 3–8 sec [158]. At around 7.6 sec, they discovered that flash integration resulted in phase delays more than twofold larger than those quantified after one hour of continuous equiluminous light exposure (despite a 3800x difference in total exposure duration). Still no effects on melatonin were observed at interstimulus intervals near ~7.6 s. The magnitude of this dichotomy is not trivial. It underscores the possibility that different flash protocols might be devised to selectively target the SCN versus the pineal gland.

The psychiatry field now sits at the precipice of a new world of possibilities for how light administration might improve the disease trajectories of those battling mental illness. At this moment, a corpus of work suggests that much finer photosyntaxes provide instruction sets for the central pacemaker, directing it to switch the timing and phase of endogenous rhythms. These instruction sets likely vary across the subjective evening and are compiled differently depending on modulatory input from other zeitgebers. They might offer the opportunity to execute complex commands that bypass the SCN through its use as a conduit or through redirection of information at the level of the retina to one of many other central areas of the brain. And they can be realized because of parallel advances in light delivery technology that have been made with LEDs, which emit nearly monochromatic light with highly precise temporal control. The warmup time for the onset of an LED—and on/off cycles—can reach nanosecond speeds. This, combined with their high energy efficiency (i.e., *luminous efficacy*, light output produced per watt of electricity invested), makes LEDs ideal for use in small medical devices [166]. In short, the software and hardware for circadian reprogramming, or reprogramming of affect or cognition, are at a convergence in their development that would allow for a realistic exploration of their potential. The possibilities for this exploration are vast and are tangibly illustrated by a brief case study of Smith-Magenis syndrome (SMS).

People with SMS, a neurodevelopmental disorder resulting from haploinsufficiency of the *RAI1* gene and bearing many similarities to autism spectrum disorder (e.g., speech and language impairment, behavioral inflexibility, motor stereotypies, and other repetitive behavior; [167–169]), exhibit disturbances in sleep and circadian rhythms that are tightly linked to inverted circadian patterns of melatonin secretion [170–175]. Individuals with SMS produce high levels of melatonin during the day (i.e., over 50 pg/ml concentration in plasma) and levels as low as 10 pg/ml during the evening, a concentration about half of that usually recorded in neurotypicals at night [171, 172]. It is an open question as to whether the phase reversal of melatonin rhythms in people with SMS results from a true inversion, or alternatively, is derived from a significant daily phase advance or delay of melatonin secretion [176]. In any event, here is a reoccurring clinical case where impaired gene expression [177] and distortions in molecular mechanisms of circadian clock function [178, 179] leave an individual differentially responsive to light and darkness' effects on melatonin [180]. In principle, this raises the possibility that patterned light can regulate melatonin more dynamically, with protocols that might step-up or down its secretion at night or those that might bypass it altogether. For the person with SMS, this might be just the tip of the iceberg for how light could be used to improve mental and physical health. Human neuroimaging experiments suggest that blue light (~470–480 nm) administration triggers activation of an attention-memory circuit that recruits the locus coeruleus, hippocampus, and frontal-parietal cortices [181]. Other experiments, at least in rodents, have demonstrated the ability of green light (525–530 nm) to alleviate pain using a pathway running from the retina down the rostral ventromedial medulla to the spinal cord [182]. By just manipulating the color spectrum of light, separate portals appear to be ratcheted open from the retinal ganglion cells to different areas of the brain. These pathways may be routes by which select neuropsychiatric problems associated with SMS (e.g., distractibility versus self-injurious behavior) are isolated for targeted rehabilitation with sleep-time phototherapy. No doubt other case studies can be made for other disorders, such as Alzheimer's disease or major depressive disorder in aging individuals.

## 6. Conclusion

We have come a long way from the days when Czeisler et al. and Lewy et al. worked to disabuse the scientific community of the notion that humans were immune to light's effects on the circadian-sleep system. What started as an outright rejection, however, has evolved into just as firm a belief that light's resetting properties are first-and-foremost dependent on exposure (i.e., reciprocity, irradiation × duration). Looking back, it is clear that this dogma grew out of the void that was left when the notion crumbled that the human circadian system vis-à-vis light was unique above all animals. It was intuitive to many that, if the human circadian system was not privileged, then it certainly was not as photosensitive as the systems studied in lower organisms such as fungi and *Drosophila* [39]. Along with the circadian flash literature, recent work continues to refute these beliefs (see [183] for a description of human resetting with a single 15 s light exposure). The fact that people are responsive to millisecond introductions of light has important implications for psychiatry. Because the effects of quick flashes do not trail-off with repeated application [158], the amount of information that can be delivered to the brain to exert change increases dramatically. The breadth of this information pool is set by the parameter space inherent to photobiology: intersecting ranges of light intensity, duration, wavelength enrichment, timing, fractionation, and photoperiod history. It is massive in scope and promises a time where it may be possible to tailor an intervention to meet the unique needs of each and every patient.

## Figures and Tables

**Box 1:**